# Supplementary material for: Molecular Epidemiology of HIV-1 in Eastern Europe and Russia
Source: Viruses. 2022 Sep 22;14(10):2099. doi: 10.3390/v14102099 (PMC9609922; doi:10.3390/v14102099)
Supplement: Supplementary file 1 [file viruses-14-02099-s001.zip › File S1 - Graphical analysis of all URF identified.pdf]

Table – Genomic maps of the *pol* gene region of unique forms

№	Sequence name	Schematic map of the genome of the studied region of the *pol* gene
**1**	**KRD846658** **URF_02/B**	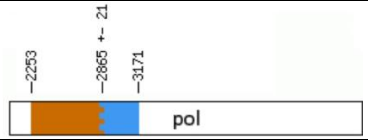
**2**	**KRD846683** **URF_A6/B**	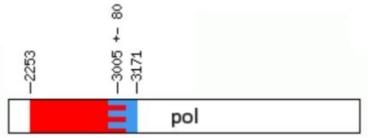
**3**	**KRD620659** **URF_A6/B**	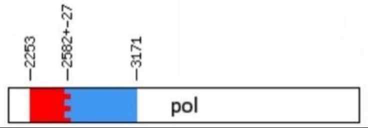
**4**	**KRD846709** **URF_A6/B**	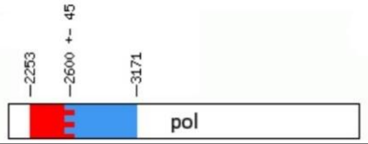
**5**	**KRD846713** **URF_A6/B**	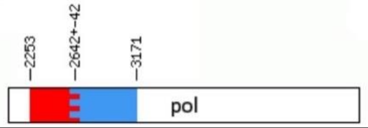
**6**	**MOS614514** **URF_B/A6**	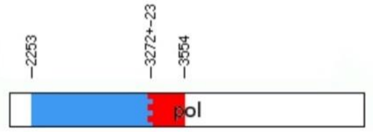
**7**	**MOS614661** **URF_A6/B/A6**	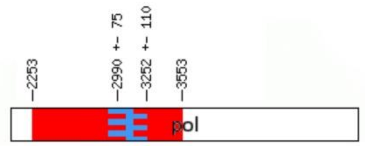
**8**	**MOS614782** **URF_A6/B/A6**	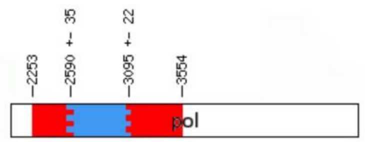
**9**	**MOS847058** **URF_63/A6/63**	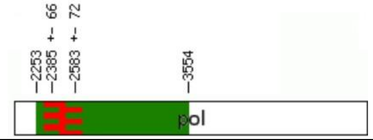
**10**	**MOS847073** **URF_B/A6**	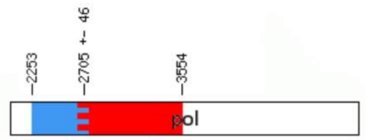

**11**	**GEO882767**<br>**URF_A/B**	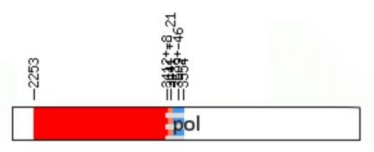
**12**	**GEO882822**<br>**URF_B/A6**	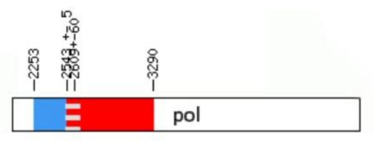
**13**	**UKR913990***<br>**URF_B/A6**	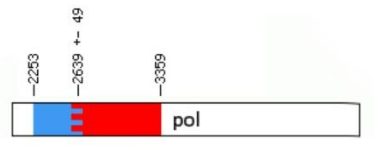
**14**	**UKR914002***<br>**URF_B/A6**	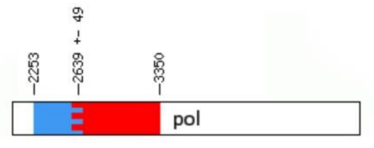

* : This URF was found in two patients; males from Ukraine that were infected by heterosexual contact. They were born in 1968 and 1996, and the virus carried the PI resistance mutation M46L.
