# Supplementary material for: Molecular Epidemiology of HIV-1 in Eastern Europe and Russia
Source: Viruses. 2022 Sep 22;14(10):2099. doi: 10.3390/v14102099 (PMC9609922; doi:10.3390/v14102099)
Supplement: Supplementary file 1 [file viruses-14-02099-s001.zip › Supplementary figures and supplementary figure legends.pdf]

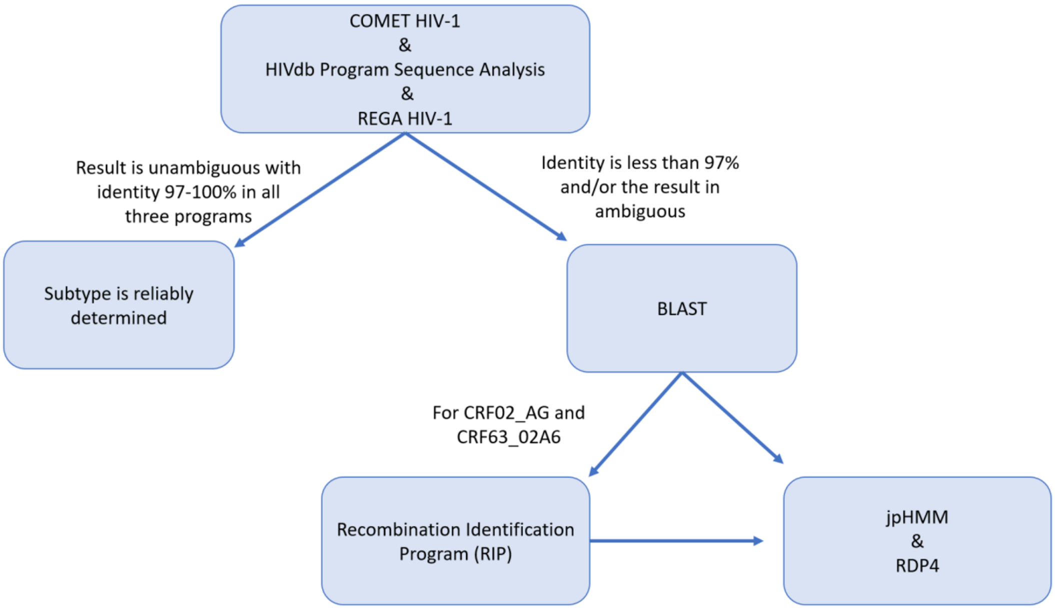

Figure S1. Flow scheme of the workflow used for the identification of circulating and unique recombinant HIV strains.

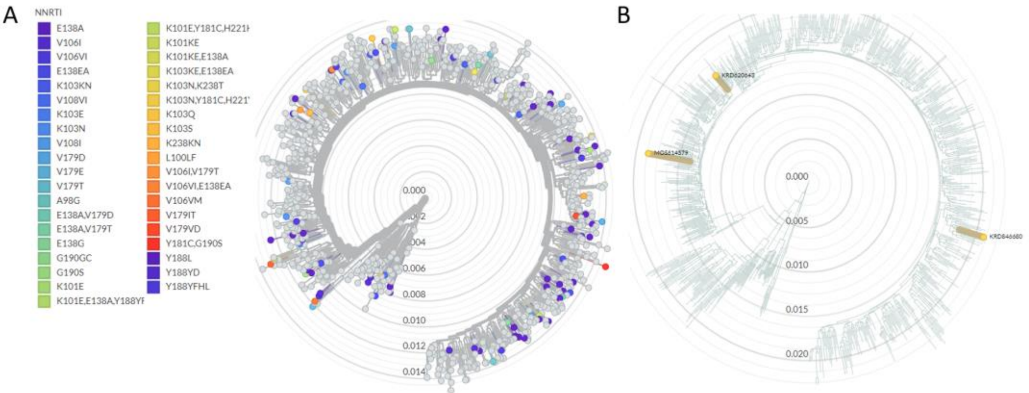

Figure S2 (A). Pre-existing NNRTI resistance mutations. The tree was rooted on a genotype D sequence (D.K0345). Phylogenetic analysis of the NRTI double and triple mutants, showing lack of relatedness between the different viruses. The tree was rooted on a genotype A1 sequence.

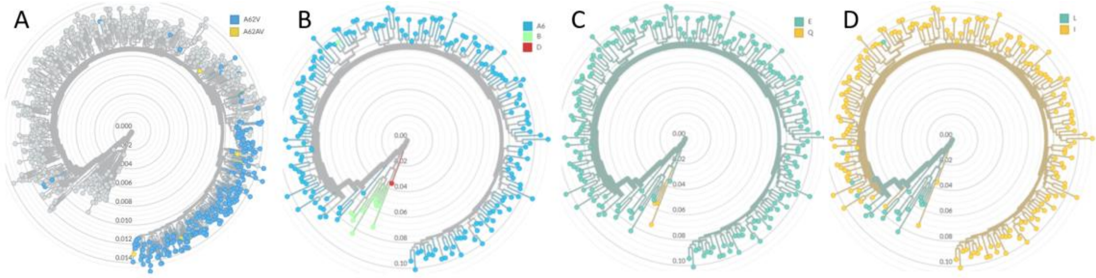

Figure S3. (A) A large cluster of viruses carrying the A62V mutation was identified. (B-D) phylogenetic analysis of the HIV-1 integrase sequences from Ukraine. The trees were coloured by (B) the genotype of the protease/reverse transcriptase genes, (C) the amino acid at position 157 (E or Q), and (D) the amino acid at position 74 (L or I). The two URFs identified in this region were excluded. All trees were rooted on a genotype D sequence (D.K0345).
