# Supplementary figures and images for: Molecular Epidemiology of HIV-1 in Eastern Europe and Russia
Source: Viruses. 2022 Sep 22;14(10):2099. doi: 10.3390/v14102099 (PMC9609922; doi:10.3390/v14102099)

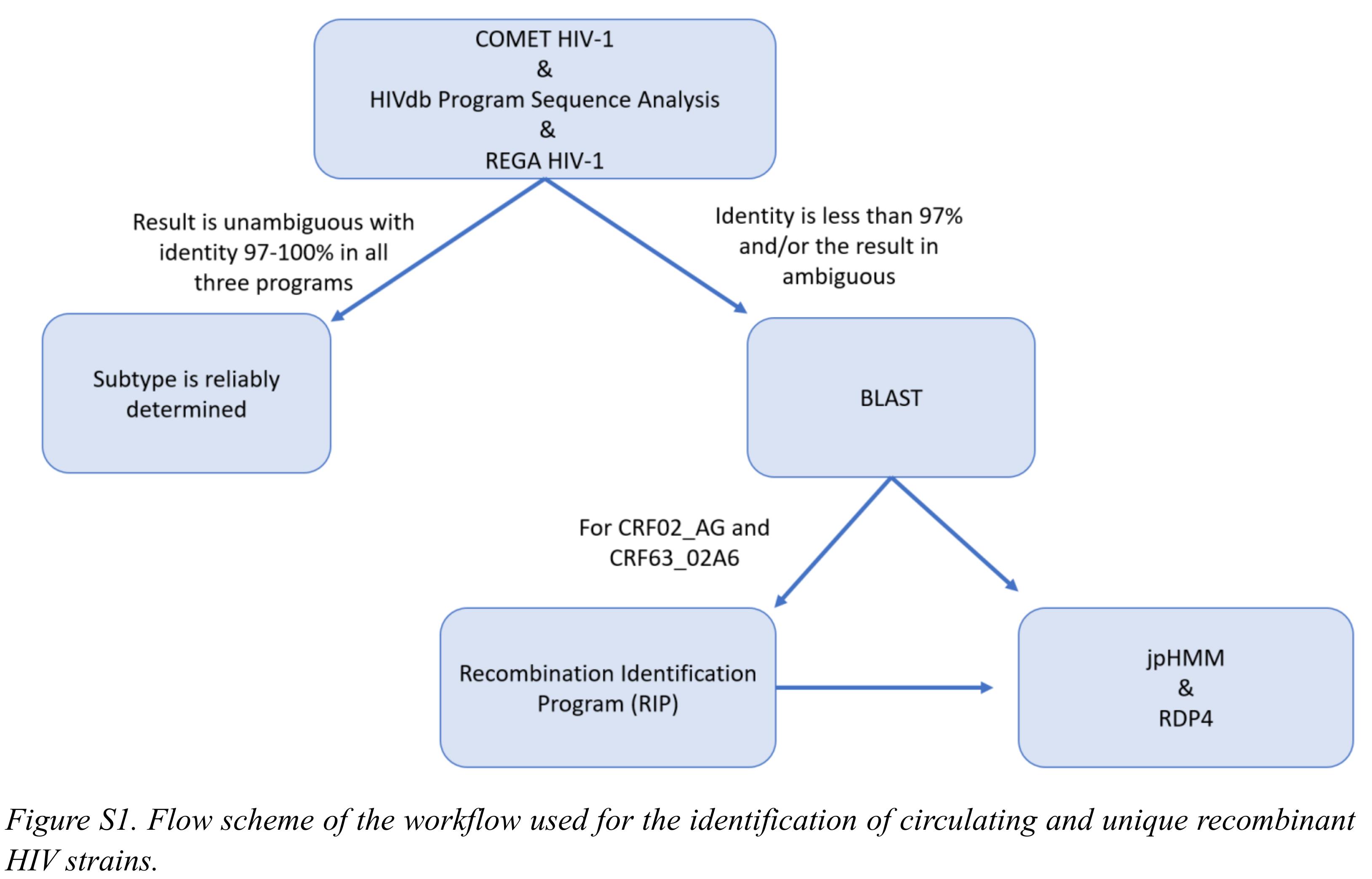

Supplement: Supplementary file 1 [file viruses-14-02099-s001.zip › Figure S1 Flow scheme of recombination analysis workflow.jpg]

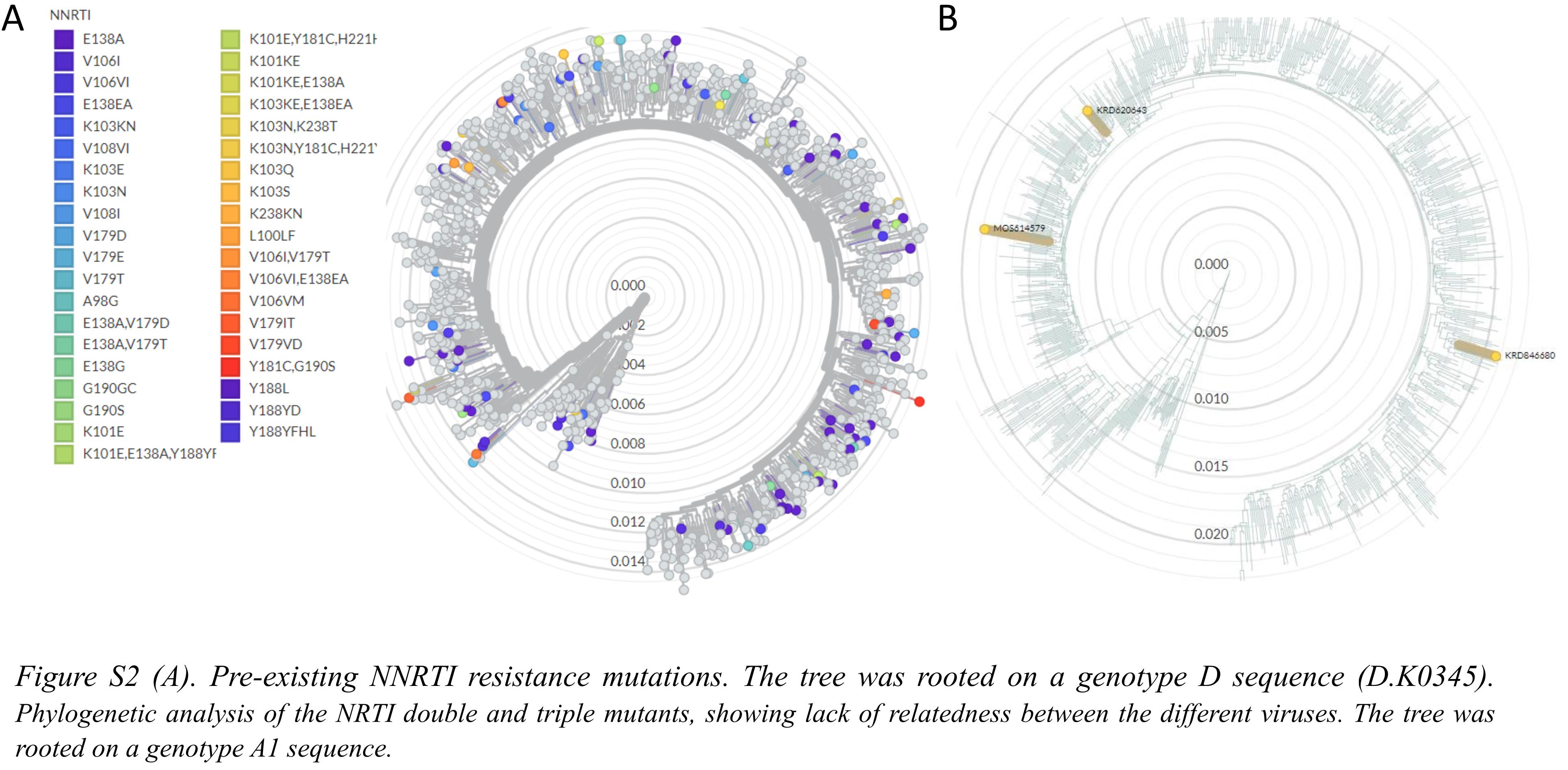

Supplement: Supplementary file 1 [file viruses-14-02099-s001.zip › Figure S2 Phylogenetic analysis of pDRMs.jpg]

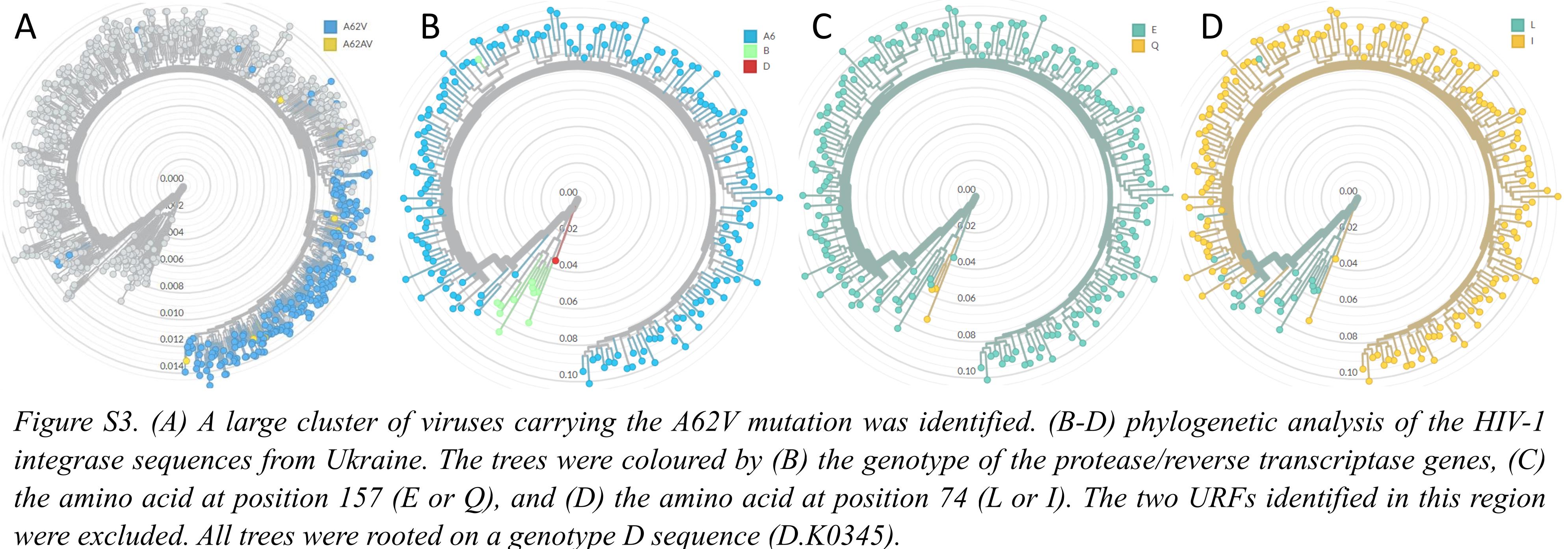

Supplement: Supplementary file 1 [file viruses-14-02099-s001.zip › Figure S3 Phylogenetic analysis A62V and Integrase sequences.jpg]
